# Supplementary material for: Prediction of Survival After Partial Hepatectomy Using a Physiologically Based Pharmacokinetic Model of Indocyanine Green Liver Function Tests
Source: Front Physiol. 2021 Nov 22;12:730418. doi: 10.3389/fphys.2021.730418 (PMC8646028; doi:10.3389/fphys.2021.730418)
Supplement: Supplementary file 1 [file Data_Sheet_1.PDF]

Supplementary Material

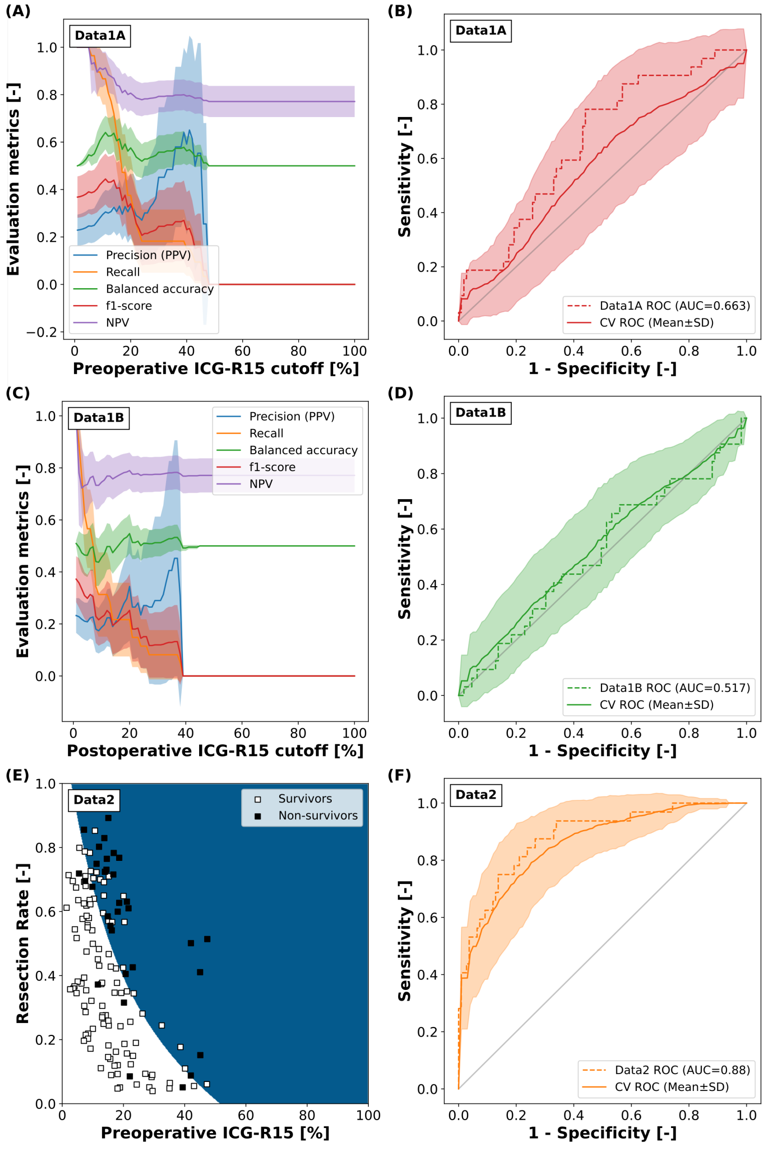

Figure S1. Classification of survivors/non-survivors after hepatectomy: **A**: Dependency of the evaluation metrics of the cross-validated classification model Data1A on the preoperative ICG-R15 cutoff (mean $\pm$ SD). PPV: positive predictive value; NPV: negative predictive value. **B:** ROC curve for the prediction of non-survival after hepatectomy using the complete dataset with cross-validation (mean $\pm$ SD) for classification model Data1A. ROC curve using the complete dataset (n=141) with cross-validation (mean $\pm$ SD) for classification model Data1A. **C**: Dependency of the evaluation metrics of the cross-validated classification model Data1B on the calculated postoperative ICG-R15 cutoff (mean $\pm$ SD). PPV: positive predictive value; NPV: negative predictive value. **D:** ROC curve for the prediction of non-survival after hepatectomy using the complete dataset with cross-validation (mean $\pm$ SD) for classification model Data1B. **E:** Decision boundary of the two-dimensional classification model Data2 based on the resection rate and $f_{cirrhosis}$ using the complete dataset. White area: predicted survivor; blue area: predicted non-survivor. **F:** ROC curve for the prediction of non-survival after hepatectomy using the complete dataset with cross-validation (mean $\pm$ SD) for classification model Data2.
